# Training providers: beyond the basics of electronic health records

**DOI:** 10.1186/1472-6963-13-503

**Published:** 2013-12-02

**Authors:** Christine E Bredfeldt, Elias Bruce Awad, Kenneth Joseph, Mark H Snyder

**Affiliations:** 1 Mid-Atlantic Permanente Research Institute, 2101 E. Jefferson St., Rockville, MD 20852, USA; 2 Mid-Atlantic Permanente Medical Group, Rockville, MD, USA; 3 KP HealthConnect - End User Support Services, Kaiser Permanente, Silver Spring, MD, USA; 4 Strategy and Operations, Clinical Information Systems, Deloitte Consulting, McLean, VA, USA

**Keywords:** Electronic medical records, Physician education, Physician behavior, Provider satisfaction, Health IT, Meaningful use

## Abstract

**Background:**

Training is a critical part of health information technology implementations, but little emphasis is placed on post-implementation training to support day-to-day activities. The goal of this study was to evaluate the impact of post-implementation training on key electronic health record activities.

**Methods:**

Based on feedback from providers and requests for technical support, we developed two classes designed to improve providers’ effectiveness with the electronic health record. Training took place at Kaiser Permanente, Mid-Atlantic States. The classes focused on managing patient-level information using problem lists and medication lists, as well as efficient documentation and chart review. Both classes used the blended learning method, integrating concrete scenarios, hands-on exercises and take-home materials to reinforce class concepts. To evaluate training effectiveness, we used a case–control study with a 1:4 match on pre-training performance. We measured the usage rate of two key electronic health record functions (problem list and medication list management) for six months before and after training. Change scores were compared using the Wilcoxon sign rank test.

**Results:**

36 participants and 144 non-participants were included in the training evaluation. Training participants were more likely to manage both medication lists and problem lists after training. Class material is now being incorporated into an enterprise-wide multi-modal training program available to all providers at Kaiser Permanente in the Mid-Atlantic States.

**Conclusions:**

Ongoing information technology training is well-received by healthcare providers, who expressed a clear preference for additional training. Training improved use of two important electronic health record features that are included as part of the Meaningful Use criteria.

## Background

Health Information Technology (HIT) has the potential to improve healthcare quality, increase patient safety, and reduce costs. Achieving that potential depends on healthcare providers being both willing and able to use the technology effectively. Despite the current high visibility of electronic health records (EHRs), many providers are unconvinced that EHRs will improve patient care and clinical outcomes [1-4]. In addition, many current providers received their medical education before information technology became ubiquitous, and are lacking both basic computer skills and the specific skills necessary to use an EHR effectively [1-3,5,6].

Training on HIT can influence providers’ willingness and ability to use EHRs effectively. Training helps providers understand how the system can be leveraged in clinical practice and introduces features and functionality with which providers may not be familiar [7]. Training is associated with improved use of advanced electronic health record features such as templates and order sets [8], and improved physician satisfaction with HIT systems [9,10].

Existing training programs are often seen as inadequate [5,11]. Studies have found that up to 94.6% of respondents claimed their ability to use the EHR could be improved [8], while 75% of respondents felt a need for additional training 5 years after EHR implementation [12]. Ongoing effectiveness HIT training may be necessary to help providers achieve mastery and a sense of control within the EHR environment.

Most studies of EHR training focus on the needs of users during the initial EHR implementation. New users may be overwhelmed at initial training, focusing on gaining basic proficiency for job function rather than efficiency and mastery. In addition, EHR software changes over time and implementation of new features may require the adoption of new workflows. In particular, as meaningful use requirements take effect, providers will be expected to be proficient at problem list and medication list management, as well as other key EHR features [13]. In order to increase proficiency on advanced EHR features, we designed and evaluated two advanced EHR classes focused on effectively managing patient-level data, efficient chart review, documentation, and entering orders.

## Methods

The study was performed at Kaiser Permanente, Mid-Atlantic States (KPMAS), using a mixed methods approach. Data extraction from the electronic health record included problem list and medication list usage and provider demographics and characteristics) (sex, age, type of practice, length of time with KPMAS) for 874 providers. In addition, a brief provider survey was administered to 54 providers who attended an EHR training class. The study was approved by the KPMAS Institutional Review Board on October 16, 2009. Informed consent was waived for training class participants as the class was delivered as part of normal operating procedures.

We identified a need for EHR effectiveness training at KPMAS through analysis of EHR support requests and questions about functionality from experienced providers who had been with KPMAS at least 2 years. KPMAS is a large (approximately 1000 physicians) integrated delivery network at which all providers use the same electronic health record for patient care. Frequent questions involved features and workflows that were introduced after the provider was initially trained on the system. Additional questions pertained to features that were introduced during providers’ initial training that were not adequately mastered. Interviews with established providers revealed a perception that newer providers had better EHR skills because of more recent training on features, workflows and tools.

The training development team consisted of a physician with advanced EHR skills (E.A.) and the senior manager of the end user support team (K.J.). Course content was developed based on four inputs: 1) analysis of common support-related questions; 2) review of efficiency content from other health care organizations; 3) review of functionality enhancements introduced over the past several years, and 4) survey of physicians who had been identified as advanced users. Advanced users were identified based on their track record of meeting organizational targets such as closing office encounters within 48 hours, timely review of labs, and same-day response to patient-initiated messages. Additionally, we consulted users who were frequent users of specific efficiency tools such as best practice advisories, short cuts, order preference lists and problem and medications lists. Content suggestions from each source were synthesized and categorized into separate classes covering related material.

**Figure 1:**
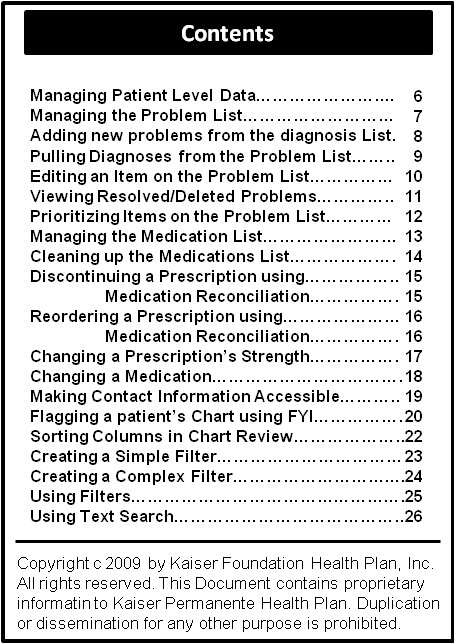
Contents of the quick reference guide that was distributed to class participants for the first class.

**Figure 2:**
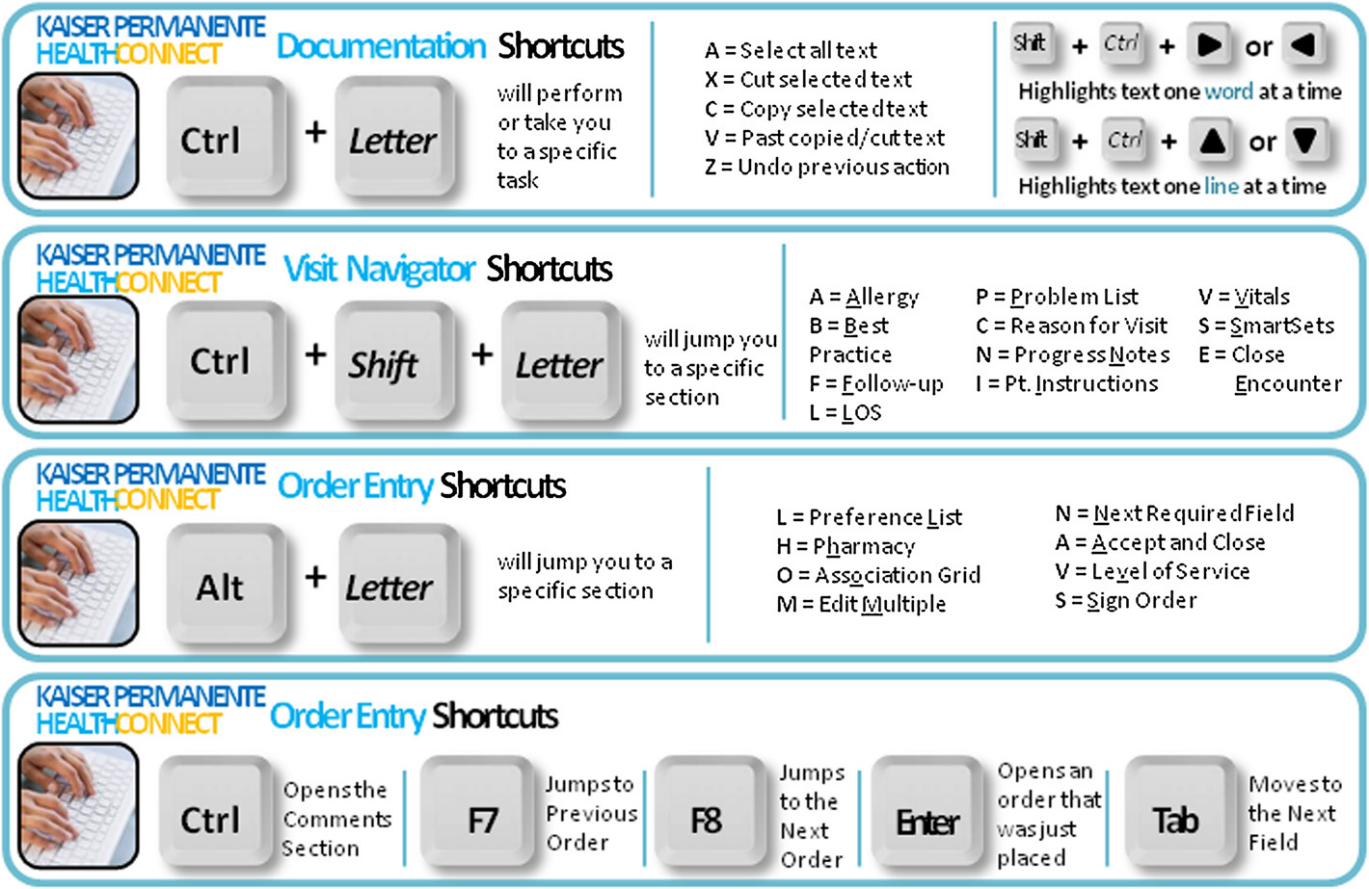
**Example of the keyboard shortcut templates.** Shortcut cards were designed to remind users of efficient key combinations to accomplish frequent tasks.

We implemented training using a blended learning format in which short lectures and demonstrations (20–40 minutes) were interspersed with hands-on directed activity [7]. We included specific hands-on exercises to allow trainees to acquire new skills while also building tools such as preference lists or personal documentation templates. These activities took place in the live EHR environment to avoid the need to duplicate the work (i.e. create the same lab results filter when back in the clinic). Ancillary materials, including a quick reference guide and keyboard shortcut template cards (see Figures 1 and 2), were provided to support post-class consolidation of learning.

We developed two separate classes. The first class covered management of patient-level data.

• Problem list management: adding, editing and updating clinically appropriate problems on the problem list, resolving and deleting problems, prioritizing entries and adding relevant comments.

• Medication list management: marking medications as chronic or active, reconciling and “cleaning up” the medication list, and reordering or changing medications.

• Patient history: adding/updating surgical and medical history, entering demographic information, and flagging charts with high priority information such as the need for an interpreter, mobility or cognitive issues, case management concerns and advance directive information.

• Efficient chart review: sorting columns, using filters and flowsheets and graphing labs.

The second class focused on the use of tools to increase efficiency.

• Documentation: basic text management tools such as spell check and autocorrect; note management tools such as the ability to find and copy previous notes.

• Efficiency tools: Developing, editing and using shortcuts to enter frequently used text including links to information from other parts of the patient’s chart, and using “SmartSets.” SmartSets provide workflow templates that group diagnosis codes, orders, patient instructions and progress note templates for common diagnoses or problems.

• Order entry: Improving efficiency and accuracy during order entry through the use of tools such as order panels and keyboard shortcuts; accurately associating orders with diagnoses from elsewhere in the patient’s chart.

• Preference list: Creating personal preference lists of frequently used orders and organizing the preference list into sections and sub-sections to improve efficiency and access.

In each class concrete patient scenarios were designed to resonate with both primary and specialty providers. Patient scenarios included a health assessment visit, a pre-operative visit, and both an initial and a follow-up visit for chronic disease. Although most training activities took place in the live EHR environment, trainees practiced patient specific exercises in a non-production version of the EHR environment to avoid altering data for KPMAS members.

After the class, participants received regular emails with “report cards” that compared their performance on key activities such as problem list management, medication list management and timely closing of encounters to other members of their specialty. These follow-up emails were designed to remind providers of key goals and help them track progress.

Each training class was led by a physician with advanced EHR skills, with an assistant to provide assistance during the hands-on exercises. Training classes lasted 4–5 hours on Saturday mornings. Each trainee had the use of a computer for hands-on exercises. Trainees received 3.5 CME credits for the first class, and 4.25 CME credits for the second class. All training participation was voluntary. Physicians were recruited by broadcast email and any physician was able to participate on a “first come first served” basis.

Evaluation: We evaluated whether training induced a change in how providers used the EHR for patient care by evaluating usage patterns in the EHR data. To be included in the analysis, providers had to have at least 100 patient visits per month for at least 6 months prior and 6 months after the training class. Controls were identified as providers who did not participate in training, but were practicing with KPMAS during the time period of interest. To ensure that data from case and control providers reflected the same time period, control providers were assigned to a specific class time period at random, and only data from that time period was analyzed.

We evaluated two outcome measures in the EHR data: the proportion of visits in which either the problem list or the medication list was modified. Problem list modification included adding or deleting problems from the problem list, or attaching comments to existing problems on the list. Modifying the medication list included marking medications as chronic, removing inactive medications, or marking the medication list as reviewed.

To evaluate whether the use of the problem list and medication list increased following training, we used a case–control methodology with a 1:4 match. Matches were based on the combined problem list and medication list management rate during the six months prior to training. By matching on pre-training performance, we reduced the impact of population differences inherent in a convenience sample. For each study provider, we calculated the change in the rate of feature use during the six months after training relative to the rate of feature use during the six months before training. Significance was measured using the Wilcoxon sign rank test.

We next evaluated whether key provider characteristics influenced the impact of training. Because our sample was smaller than is necessary for robust regression (N = 36), we evaluated each of six characteristics individually. For each of four continuous variables (age, length of time with KPMAS, number of monthly encounters and baseline performance) we assessed the Pearson correlation of the variable with the outcome variables. For the remaining two categorical variables (sex, Primary vs. Specialty care provider) we used the Student’s *t*-test to estimate significance.

## Results

We developed two classes designed to train providers to be more effective when using the EHR: one class focused on chart review and managing patient level data, and the other focused on accurate and efficient documentation and order entry. A total of 43 providers participated in the first class and 45 providers attended the second class. Participants included 39 Primary Care Providers and 15 Specialists. 34 providers attended both classes, for a return rate of 79%.

Participants were asked to complete a one-page evaluation of the program consisting of four open-ended questions:

1. List one or two things you will do differently or will share with your colleagues as a result of attending this training.

2. Please list one thing the speakers did well.

3. Please list one thing that could be improved.

4. Additional comments/Suggested future topics.

Two major themes emerged in the responses. First, class participants felt the training classes should be offered more frequently and on a wider range of topics. The second theme revolved around the use of hands-on exercises in the training class. Class participants indicated that the hands-on exercises were the most useful portion of the class, and they appreciated the ability to build things in class that could be used in the clinic.

**Figure 3:**
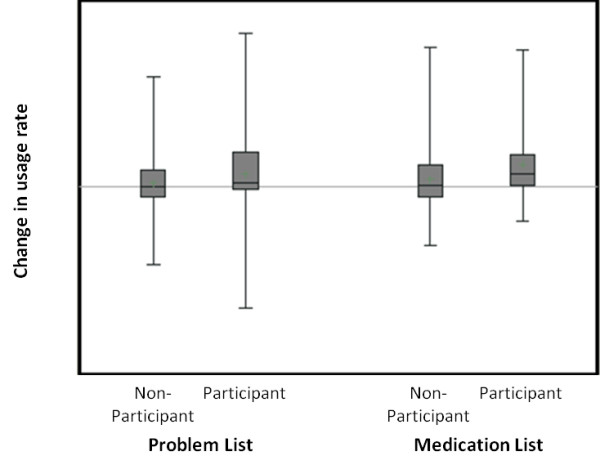
**Effect of training on use of problem lists and medication lists.** Each box and whisker represents the distribution of performance scores for either problem list usage (left) or medication list usage (right). The box represents the inter-quartile range, while the upper and lower lines indicate the extreme values. The horizontal line in each box indicates the mean.

We measured performance on two skills covered by the training classes: modifying the problem list and modifying the medication list. 36 training participants and 144 non-participants met the criteria for inclusion in the evaluation. The box and whisker plot in Figure 3 illustrates the change in performance after training for both training participants and non-participants. Training participants were more likely to increase their use of both the medication list (p < 0.05, Wilcoxon sign rank test) and the problem list during the six months after training, although the improvement in problem list use did not reach significance (p = 0.06, Wilcoxon sign rank test). On average, participants increased their use of the problem list from 22% of visits to 24% of visits, a 2 percentage points of usage. Training participants also increased their use of the medication list 4 percentage points of usage, from 41% of visits to 45% of visits.

We next evaluated the relationships between 6 provider variables and the observed changes in problem list and medication list management among training participants to determine if the impact of training was dependent on provider demographic or practice characteristics. Table 1 shows the magnitude and significance of the effects of six provider characteristics. None of the provider characteristics had a statistically significant impact on post-training performance for either problem list management or medication list management. However the results did suggest that baseline performance on problem list management may be negatively correlated post-training improvements, although the relationship was not strong enough to be significant in our small sample (p < 0.1).

**Table 1:** Relationship between provider characteristics and outcome measures

**Continuous variables**	**Problem list management**		**Medication list management**			
	**Correlation coefficient**	**p value**	**Correlation coefficient**	**p value**		
Age	−0.12	p = 0.48	0.20	p = 0.24
Length of time with KPMAS	−0.16	p = 0.34	0.21	P = 0.22
Number of encounters during baseline period	0.06	p = 0.72	0.00	p = 0.99
Baseline performance	−0.31	**p = 0.07**	−0.21	p = 0.21
**Categorical variables**	**Difference score**	**p value**	**Difference score**	**p value**
Sex	0.02	p = 0.47	0.03	p = 0.34
Type of provider (primary or specialty care)	0.04	p = 0.13	−0.02	p = 0.38

## Discussion

Well-timed and carefully planned training can play a critical role in successful HIT implementations [14-17]. However, there have been few studies of the role of training in maintaining and upgrading technology competencies. HIT implementations undergo frequent upgrades involving new and modified features. In addition, as meaningful use criteria for EHR incentive programs evolve, providers will need to expand their information technology competencies to encompass the new skills. This study demonstrated that ongoing training may increase the use of medication lists and problem lists, although the small sample size meant that some effects did not reach significance. A larger study is warranted to determine if ongoing training may be useful to help providers to maintain a high level of competency and efficiency across a wide array of tasks.

We developed and delivered training to improve providers’ skills at daily workflow tasks. The content was developed primarily based on questions and problems identified by providers during their daily activities. The course content focused on high-impact workflows that improve communication between providers and support patient safety and healthcare quality [18,19]. We also focused on improving the efficiency of workflows for documentation and entering orders in order to help providers chart more efficiently and effectively.

We experienced higher than anticipated interest in the training classes. Interested providers included physicians, nurse practitioners and nurses. The positive response suggests there may be an underlying interest and need for ongoing EHR training, at least within the KPMAS environment. This finding is consistent with previous work that found strong support for the positive role of training on EHR tools [14,20]. Factors critical to the training program’s success were the use of hands-on exercises that were relevant to providers’ practice patterns [7], a live EHR system to allow providers to build tools they could use when they returned to the clinic, and the use of ancillary materials. The use of post-class performance reporting also served to keep participants engaged after they completed the class and helped remind them of class lessons.

We also experienced several challenges related to the higher-than-anticipated demand. The high level of interest from relatively basic users required us to add additional staff to limit the impact of beginners on the class. Future training opportunities will need to carefully assess the size and the current proficiency level of trainees to provide adequate staff and training time. Alternative delivery methods such as online webinars, in-clinic one-on-one training and team-based training may also increase training opportunities [21].

We found that training improved two key EHR skills that are integral to stage 1 and stage 2 meaningful use criteria: medication list management and problem list management. Although there are no commonly accepted rates for problem list and medication list management, KPMAS policy is that both the medication list and the problem list should be reviewed at every visit. While not all reviews are likely to result in modifications, the pre-training average use rates in this study of 22% and 41% for problem list and medication list use respectively indicated there was considerable room for improvement. We found that although medication list management showed training-related improvements regardless of pre-training proficiency, there was a trend indicating an inverse relationship between problem list management and pre-training proficiency. This difference likely reflects an underlying difference in how problem lists and medications lists are used in patient care. Changes to the problem list generally reflect onset or resolution of chronic health concerns, and therefore may not require modification at visits where the focus is on acute care. However, the medication list contains entries for both chronic and acute problems, and needs to be updated at virtually every visit in order to ensure accuracy. In this study, trainees who are proficient at problem list management may have already reached the functional ceiling, leaving no room for improvement. In contrast, while some training participants were very practiced at medication list management, there was still room for improvement. Future research should more clearly identify appropriate target rates for problem list and medication list management, in order to better target EHR training.

There are some issues to consider in applying the lessons learned at KPMAS to other healthcare institutions. The training was developed for a non-specific outpatient setting including both primary care and specialty care. The training elements used in this setting may not be as appropriate in an inpatient setting. In addition, the training may have been more effective if it were specialized type of practice (i.e. primary care or specialties). In addition, the training was developed for a large, integrated delivery care network where all providers use the same electronic health record tools and consistent workflows. Developing efficiency training modules for small provider groups could be prohibitively expensive. However, cost sharing among practices using the same EHR system could provide viable training opportunities for such groups.

## Conclusions

Overall, we found the providers valued advanced training on EHR tools and workflows, to the extent that they were willing to participate on Saturdays and return for additional content. Training was related to a small, but significant increase in the use of key EHR capabilities included in meaningful use criteria.

## Abbreviations

EHR: Electronic health record; HIT: Health information technology; KPMAS: Kaiser Permanente, Mid-Atlantic States.

## Competing interests

The authors have no financial or non-financial competing interests related to the subject matter discussed in this manuscript.

## Authors’ contributions

CB, EA, KJ, and MS contributed to the study design. EA and KJ designed and developed the training. EA delivered the training. CB wrote the original draft and performed the literature review. All authors provided comments on the manuscript. All authors have read and approved the final manuscript.

## Pre-publication history

The pre-publication history for this paper can be accessed here:

http://www.biomedcentral.com/1472-6963/13/503/prepub
